# Defibrotide improved the outcome of monocrotaline induced rat hepatic sinusoidal obstruction syndrome

**DOI:** 10.1186/s12876-022-02523-3

**Published:** 2022-12-16

**Authors:** Zhenli Liu, Shan Liang, Xinhuan Wei, Xiaofei Du, Jing Zhang

**Affiliations:** The Third Unit, Department of Hepatology, Beijing Youan Hospital, Capital Medical University, No. 8, Youwai Xitoutiao Street, Fengtai District, 100069 Beijing, China

**Keywords:** Hepatic sinusoidal obstruction syndrome, Monocrotaline, Defibrotide, Low molecular weight heparin

## Abstract

**Background and aim:**

Pyrrolizidine alkaloids (PA) induced hepatic sinusoidal obstruction syndrome (HSOS) occurred worldwide and the mortality rate remained high because there were no specific therapies. Defibrotide was effective for HSOS following hematopoietic stem cell transplantation. But the pathogenesis of the two types of HSOS were not equivalent. The purpose of this study was to see if defibrotide was also effective in PA induced rat HSOS.

**Methods:**

First we improved rat HSOS model by using higher dose (230 mg/kg) of monocrotaline (a kind of PA) as the dose of median lethal dose. So drug effectiveness could be assessed by survival time. Next, male SD rats were divided into 5 groups. They were control group, model group, low dose low molecular weight heparin (LMWH) treatment group, high dose LMWH treatment group and defibrotide treatment group. Rats’ survival time, liver function, white blood cell count and cytokines were compared among the groups. The DeLeve score was used to assess the severity of liver pathology.

**Results:**

The model group exhibited typical liver pathology of HSOS, such as hepatic sinus dilation, congestion, endothelial injury of central lobular vein, coagulative necrosis of hepatocytes and fibrin deposition in the subendothelial. The pathologic characteristics indicated that the model was built up successfully. The survival rate was significantly higher in defibrotide group (81.8%) than model group (43.7%), while the survival rates were similar in the two LMWH groups (62.5% and 75%) and model group. The survival time only be prolonged by defibrotide (P=0.028) but not LMWH (P>0.05). DeLeve score was improved most in the defibrotide group than the two LMWH groups (both P<0.01). Changes in DeLeve score, liver function, plasma level of tumor necrosis factor α and plasminogen activator inhibitor-1 exhibited the same trends.

**Conclusion:**

Defibrotide could improve the outcome of monocrotaline-induced rat HSOS indicating that defibrotide might be a better choice than LMWH in clinical practice.

## Introduction

Hepatic sinusoidal obstruction syndrome (HSOS, previously named hepatic veno-occlusive disease or HVOD), was first discovered in patients who underwent hematopoietic stem cell transplantation (HSCT). The typical manifestations were hepatomegaly, ascites, right upper quadrant pain, jaundice, and abnormal liver transaminases [2]. The main pathogenesis was sinusoidal endothelial cells (SECs) injured by hemotherapy or radiotherapy during HSCT [1]. HSOS was an important cause of death after HSCT.

Besides HSCT, HSOS could be caused by a variety of other factors, such as chemotherapy drugs, immunosuppressants, monoclonal antibodies, and herbal medicine [1]. There were more than 6000 plants containing pyrrolizidine alkaloids (PA) and half of them were hepatotoxic [2, 3]. PA caused more than 17,000 cases of HSOS worldwide and made up the major cause of this syndrome [2, 3]. The mortality rate ranged from 30 − 67% and some patients survived with sequelae such as portal hypertension or cirrhosis [3].

Defibrotide had antithrombotic, fibrinolytic, and angiogenic properties. It was the only drug which was approved to prevent and treat HSOS following HSCT [4, 5]. Low molecular weight heparin (LMWH) was recommended as first-line therapy for pyrrolizidine alkaloids (PA) induced HSOS by a Chinese expert opinion though there were not sufficient evidences [6–8] and the mortality rate was still very high. In the study, we planned to compare the efficacy of defibrotide and LMWH in an improved rat HSOS model in order to see whether defibrotide was also effective for PA induced HSOS and better than LMWH.

## Materials and methods

### Animals

Male SD rats were supplied by Beijing Charles River Experimental Animal Technology Co., Ltd. They were kept in a 12-hour dark/light cycle at a temperature of 18–25 °C and a humidity of 55%. The study was approved by the Animal Experimental Ethics Committee of Capital Medical University.

### Improve a rat model of monocrotaline (MCT) induced HSOS

There were several acute or chronic HSOS models previously. The toxic agents were mainly MCT [9] or retrorsine [3], both were one kind of PA. However, there was not any model suitable for assessing drug efficacy by increasing survival time [10, 11]. We improved a rat HSOS model by using median lethal dosage(LD_50_) in this study. First, 20 male rats were randomized into 4 groups using random number table. MCT (Medchemexpress LLC.) were garaged at a single dose of 500, 450, 400, and 350 mg/kg after starvation for 12 h. The Dm of MCT was found to be 400 mg/kg. Second, another 20 male rats were randomly divided into 4 groups and being garaged by MCT at the doses of 220 mg/kg, 180 mg/kg, 140 mg/kg, and 100 mg/kg, respectively. The Dn dose was discovered to be 140 mg/kg. Finally, six dosages were used to determine LD_50_.They were 400 mg/kg, 320 mg/kg, 256 mg/kg, 204 mg/kg, 163 mg/kg and 131 mg/kg. Each group had 10 rats. Within 7 days, there were 10, 8, 7, 4, 1, 1 rats died in the six groups respectively. LD _50_ was calculated to be 229.31 mg/kg [95%CI (201.88 mg/kg, 259.97 mg/kg)]. In the following study, the HSOS model was established using MCT garaged at a single dose of 230 mg/kg.

### Drug dose conversion between human and rat

If the dose of the drug is W mg/kg for patients, the dose for rats should be W mg/kg ×70 kg×0.018/200 g. According to the formula, the dose of defibrotide should be 50 mg/kg. We dissolved defibrotide in saline at a concentration of 20 mg/ml. The high and low doses of LMWH were 400IU/kg/d and 200IU/kg/d, respectively.

### Animal grouping and model set up

**Fig. 1:**
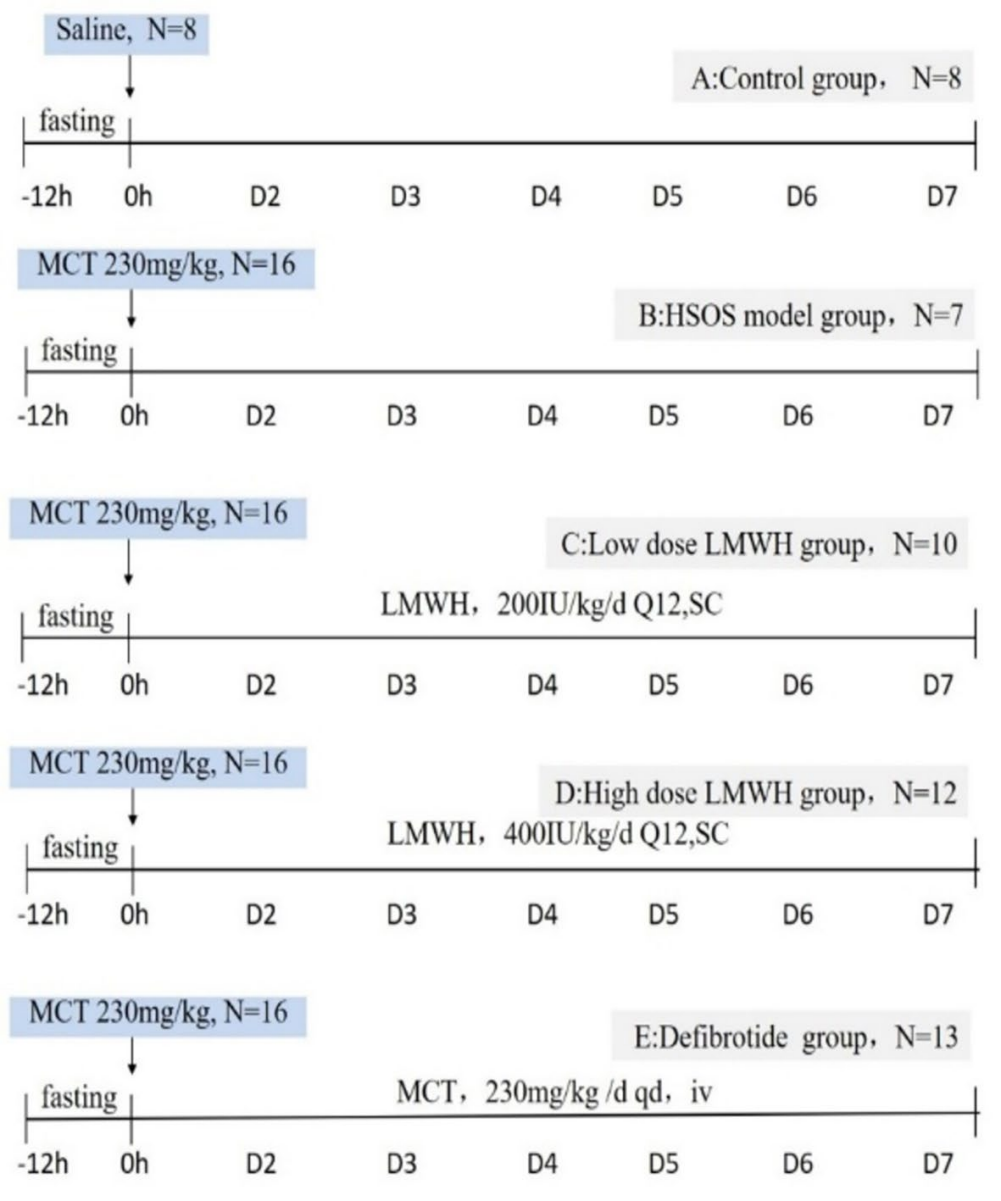
Experimental protocol for assessment of effectiveness of defibrotide for hepatic sinusoidal obstruction syndrome. MCT, monocrotaline; LMWH, low molecular weight heparin.

A total of 72 male SD rats weighing 200-250 g were randomly assigned to one of five groups. Group A (control group) had eight rats which were garaged once with 1 ml PBS at the baseline. Sixteen rats in group B (HSOS model group) were garaged once with 230 mg/kg MCT at the baseline. The other 16 rats in group C (low dose LMWH treatment group) were treated with LMWH at a dose of 200IU/kg/d (Q12h, SC)in addition to MCT garage. The treatment of rats in group D (high dose LMWH treatment group) was similar to group C but the dose of LMWH was 400IU/kg/d. Sixteen rats in group E (defibrotide treatment group) were injected with defibrotide through tail venous daily at the dose of 50 mg/kg/d in addition to MCT garage. At the end of day 7, there were 8, 7, 10, 12 and 13 rats survived in group A to E and the survival rates were 100%, 43.8%, 62.5%,75.0% and 81.3%, respectively (Fig. 1).

### Blood and liver sample collection and testing

Rats were starved for 12 h and had access to water freely. On the day 0, 1 ml saline or MCT, was garaged at 9 AM. The survived rats were sacrificed on the 7^th^ day at 9 AM. The rats were weighed and anesthetized with 2% pentobarbital sodium at the dose of 2 mg /kg. Blood sample was drawn from the abdominal aorta to test ALT, AST, bilirubin, white blood cell (WBC), plasma tumor necrosis factor-α (TNF-α), and plasminogen activator inhibitor-1 (PAI-1) (ELISA kit, provided by Dade Behring Inc.). Rat liver was taken out and the left lateral lobe of the liver was fixed immediately in 4% paraformaldehyde. After hematoxylin and eosin (H&E) staining and Masson staining, a single pathologist evaluated the histology of HSOS. The severity of HSOS was quantified using the DeLeve score system [9]. In brief, eight pathology lesions were described, weighted, and added up. The lesions were:1) endothelial damage of the central venule (CV); 2) coagulative necrosis of hepatocytes; 3) subendothelial hemorrhage of the CV; 4) sinusoidal hemorrhage; 5) subendothelial fibrosis of the CV; 6) adventitial fibrosis of the CV; 7) inflammation of the CV, and 8) lobular inflammation. The lesions were classified as early HSOS (if CV fibrosis was grade 0–1) and late HSOS (if CV fibrosis was grade 2–3). The score of early HSOS was calculated by adding the individual scores for lesion 1, lesion 2, and lesion 3 or 4 together (the higher score was selected and added on). The score of late HSOS was calculated by adding score of lesion 1 with score of lesions 3 or 4 (the higher score was selected and added on), and lesion 5 or 6 (the higher score was selected and added on). No matter the stage of HSOS, 2 or 3 points were classified as mild injury, 4 to 6 points as moderate injury, and 7 to 9 points as severe injury.

### Statistics

Descriptive values were expressed as mean ± standard deviation (SD). Student t-test or Mann-Whitney-Wilcoxon test was used to assess continuous variables according to value distributions. Categorical variables were summarized using frequencies and percentages, and data were compared using Pearson’s Chi-square or Fisher’s exact tests as needed. The χ^2^ test was also used if indicated. The Statistical Package for Social Science for Windows, version 19.0 (SPSS Inc., IBM, New York) was used for data analyses. All comparison tests between two groups were 1-tailed with a 95% confidence interval. The survival differences were analyzed using the Kaplan-Meier method, with the Kaplan-Meier estimates being compared using a log-rank test. The statistical significance was set at the p-value of < 0.05.

## Results

### The pathology characteristics of MCT induced HSOS

**Fig. 2:**
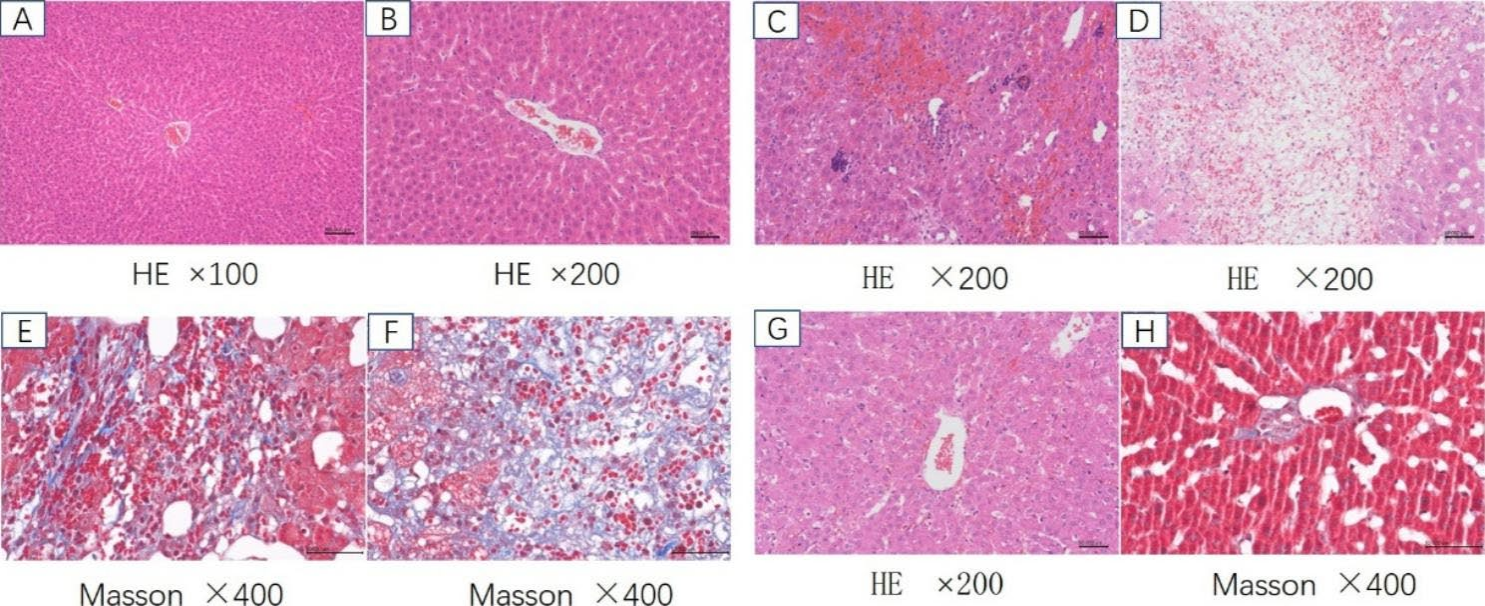
The pathology results of each group. Figure 2A and 2B: Control group, the structure of hepatic lobules was intact, and there was no degeneration, necrosis, inflammatory cell infiltration or fibrin disposition. Figure 2C: model group, sinusoidal hemorrhage. Figure 2D: model group, coagulative necrosis. Figure 2E and 2F: model group, bilirubin deposition in the subendothelial, adventitia of hepatic sinuses and central veins. Figure 2G: defibrotide group, sinusoidal hemorrhage but much less than in Fig. 2C. Figure 2H: defibrotide group, slight subendothelial and advential fibrosis much less than in Fig. 2E and 2F.

The liver histology of group A (control group) was normal under light microscopy, as shown in Fig. 2A and 2B. In the model group (group B), the histology exhibited typical lesion of HSOS, such as hepatic sinus dilation, congestion, endothelial injury of the central lobular vein, coagulative necrosis of hepatocytes, and infiltration of inflammatory cells (Fig. 2C and 2D). Masson staining showed fibrin deposition in the subendothelial, adventitia of hepatic sinuses and central veins (Fig. 2E and 2F).The pathology findings indicated that the HSOS model had been successfully established.

### The effectiveness of LMWH and defibrotide on HSOS assessed by liver pathology

**Fig. 3:**
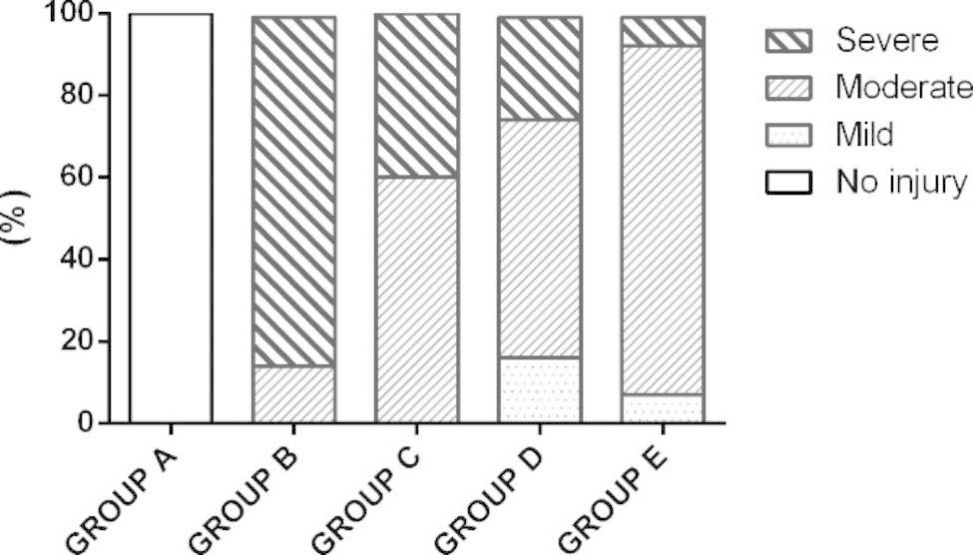
Severity of liver injury assessed by DeLeve score in each group. Group A: control group; Group B: HSOS model group; Group C: low dose LMWH group; Group D: high dose LMWH group; Group E: defibrotide group.

According to the DeLeve score system, the liver pathology of each survived rat was assessed and classified (Table 1). Liver injuries in groups C, D, and E were much less than in group B (Fig. 2G and 2H). Six rats in group C were moderate injury and 4 rats were severe injury. The typical lesions were sinusoidal hemorrhage and adventitial fibrosis of the CV. In group D, there were 2, 7 and 3 rats with mild, moderate and severe injury, respectively. In group E, the numbers were 1, 11 and 1, respectively. (Fig. 3)

**Table 1:** Severity of liver injury assessed by DeLeve score (n (%))

Group (Survived No.)	No	Mild	Moderate	Severe
A (n = 8)	8 (100%)	0 (0)	0 (0)	0 (0)
B (n = 7)	0 (0)	0 (0)	1 (14.3)	6 (85.7)
C (n = 10)	0 (0)	0 (0)	6 (60.0)	4 (40.0)
D (n = 12)	0 (0)	2 (16.7)	7 (58.3)	3 (25.0)
E (n = 13)	0 (0)	1 (7.7)	11 (84.6)	1 (7.7)

The DeLeve score of group C, D, and E were all significantly lower than group B (all P<0.001). The DeLeve score of group E were significantly lower than group C and D (both P<0.001)

Group A: control group; Group B: HSOS model group; Group C: low dose LMWH group; Group D: high dose LMWH group; Group E: defibrotide group

In brief, the severity of the liver injury was group B>C>D>E>A (all P<0.01). The results indicated that defibrotide was more effective than low dose and high dose LMWH for MCT-induced HSOS.

### Comparison of survival time among groups

**Fig. 4:**
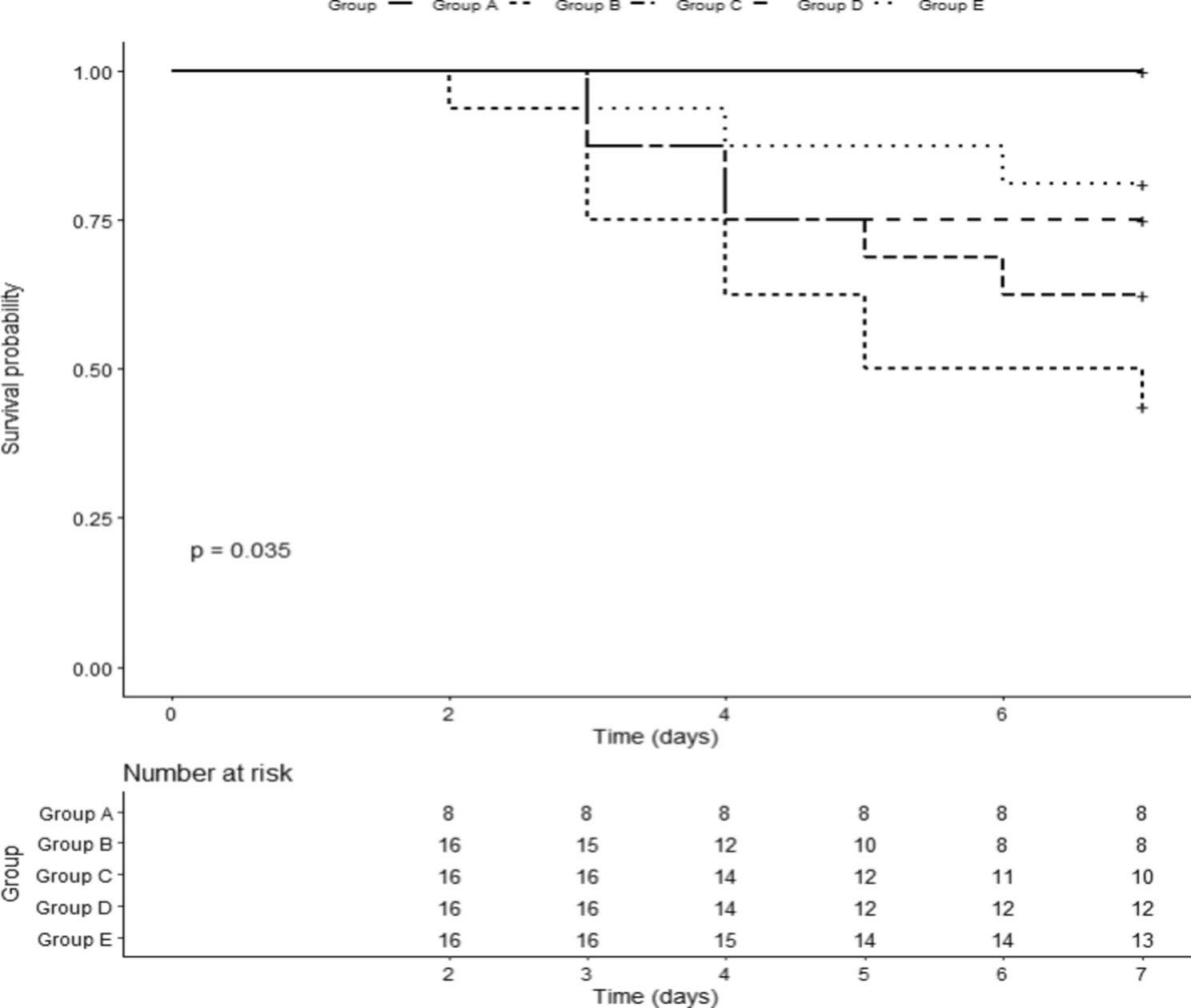
The survival curve of each group. Group A: control group; Group B: HSOS model group; Group C: low dose LMWH group; Group D: high dose LMWH group; Group E: defibrotide group. The survival time between group E and B was significantly different (P = 0.028). The survival time between group B, C and D was similar (P>0.05).

In the study, 22/72 rats died within 7 days. The 7-day survival rate in A-E groups was 100%, 43.7%, 62.5%, 75% and 81.8%, respectively. Kaplan-Meier analysis showed that defibrotide (group E) significantly prolonged the survival time than group B (P=0.028), while there were no differences between group B and group C or D (Fig. 4).

### Comparison of liver functions and WBC count among different groups

Liver function and WBC count were tested in the surviving rats. The parameters increased paralleled from groups A, E, D, C, and B. The results indicated a better effect of defibrotide than LMWH (Table 2).

**Table 2:** Comparison of liver function and WBC count among groups

Group (Survived No.)	ALT (U/L)	AST (U/L)	Bilirubin (µmol/L)	WBC (10^9^/L)
A (n = 8)	47.4 ± 3.7	141.5 ± 9.3	0.88 ± 0.11	6.0 ± 0.9
B (n = 7)	143.6 ± 11.8*	478.3 ± 18.1*	18.72 ± 1.50*	11.2 ± 0.5*
C (n = 10)	122.3 ± 3.7*	396.3 ± 6.5*#	14.32 ± 3.71*#	10.6 ± 0.8*
D (n = 12 )	64.4 ± 4.5*#	220.3 ± 5.3*#	5.55 ± 3.60*#	8.1 ± 0.2*#
E (n = 13 )	60.2 ± 4.9*#	195.3 ± 3.7*#	5.80 ± 2.60*#	8.3 ± 0.4*#

注: *P < 0.05, compared to group A; #P < 0.05, compared to group B

Group A: control group; Group B: HSOS model group; Group C: low dose LMWH group; Group D: high dose LMWH group; Group E: defibrotide group

### Plasma level of TNF-α and PAI-1 in different groups

Plasma TNF-α levels in groups B, C, and D were significantly higher than group A, but there were no significant differences between group E and group A. Plasma PAI-1 was significantly higher in groups C, D, and E than group A and lower than group B (Table 3).

**Table 3:** Plasma level of TNF-α、PAI-1 in different groups

Groups (Survived No.)	TNF-α (pg/ml )	PAI-1 (ng/L )
A (n = 8 )	101.8 ± 11.5	386.4 ± 12.8
B (n = 7 )	373.4 ± 14.1*	813.4 ± 25.9*
C (n = 10 )	258.1 ± 12.4*#	747.6 ± 27.1*#
D (n = 12 )	152.3 ± 17.3*#	512.0 ± 23.4*#
E (n = 13 )	107.6 ± 11.5#	446.7 ± 32.0*#

*Compared to group A, P < 0.05, #Compared to group B, P < 0.05

Group A: control group; Group B: HSOS model group; Group C: low dose LMWH group; Group D: high dose LMWH group; Group E: defibrotide group

## Discussion

In this study, we discovered that the effect of defibrotide was better than LMWH on MCT induced HSOS, supported by increasing of survival time, improvement of histology, liver function and cytokines. As we knew, this was the first attempt to evaluate defibrotide effectiveness on MCT induced HSOS and might provide new choice for clinical treatment.

HSOS was a common complication after HSCT. The main cause was injury of sinusoidal endothelial cells (SECs) during hemotherapy or radiotherapy. The mechanisms included glutathione depletion, nitric oxide depletion, increased intrahepatic expression of matrix metalloproteinases and vascular endothelial growth factor and activation of clotting factors [9]. In such circumstances, SECs were activated and characterized by increase of von Willebrand factor expression, platelet adhesion and prothrombotic state. While injured, SECs rounded up, favored the appearance of gaps in the sinusoidal barrier, facilitated the egress of red blood cells, WBC and cellular debris into the space of Disse and in turn dissected the endothelial lining. The sloughed sinusoidal lining embolized downstream and obstructed sinusoidal flow which leading to the obstruction of the hepatic sinusoids. The clinical manifestations were characterized by enlargement of liver and spleen, ascites and liver injury. If the injury continued or not be treated in time, hepatocytes necrosis would progress to liver failure [2, 5]. The mortality rate of the severe type HSOS was higher than 80% [5].

The pathology and clinical manifestation of PA-induced HSOS was similar to the HSOS following HSCT, but the pathogenesis of SEC injury was different [10]. The active metabolite of PA, monocrotaline pyrrole, bounded covalently to the actin microfilaments of SEC. Depolymerization of the F-actin led to actin cytoskeleton disassemble and SEC round up. Overexpression of matrix metalloproteinase-9 led to breakdown of the extracellular matrix in the space of Disse and accelerating dehiscence of the endothelial cells [10]. Once SEC was injured, the consequent damage of the liver developed successively which was similar to HSOS following HSCT.

In the past, several drugs have been attempted to treat or prevent HSOS following HSCT. Tissue plasminogen activator (t-PA) had anticoagulant and solubilizing properties, but could not increase day 100 + survival rate [11, 12]. Glucocorticoids could relieve inflammation, improve blood supply to the liver, inhibit hepatic stellate cell activation and decrease fibrin deposits. Beihany et al. [13] showed that patients who responded to methylprednisolone had a higher survival rate (58%) than those who didn’t (10%). Myers et al. reported similar results [14]. But methylprednisolone was not recommended by guidelines due to limited evidences. The only drug recommended by guidelines was defibrotide.

Defibrotide was a sodium salt of complex single-stranded oligodeoxyribo-nucleotides derived from porcine mucosal DNA. Defibrotide has been proved to stabilize and protect endothelial cells against activation, with properties of profibrinolytic, antithrombotic, anti-ischemic, anti-inflammatory and antiadhesive activities, without obvious systemic anticoagulant side effects [15]. The mechanisms included stimulating release of thrombomodulin, tPA, prostacyclin and prostaglandin E2, decreasing thrombin generation, tissue factor expression, PAI-1 release and endothelin activity, etc. [16]. In the phase 3 study, defibrotide significantly increased the day 100 + survival rate (38.2% vs. 25.5%) and the complete response (CR) rate (25.0% vs.12.5%) than the control group [17]. A meta-analysis [15] showed that the 100 + day survival rate and CR rate of HSOS following HSCT treated by defibrotide were 58% and 57%. The ratios were 44% and 39% for severe patients, respectively. Usually, the 100 + day survival rate and CR rate without defibrotide treatment were less than  20% and  31%, respectively. Based on these results, defibrotide was suggested to prevent and treat HSOS following HSCT by the European Association for the Study of the Liver [1] and the Middle East/North Africa regional consensus [4]. But there was no research on it’s effect onMCT-induced HSOS.

There were few studies on the treatment of PA induced HSOS. Sorafenib [18] and regorafenib [19] were only tested in animal experiments. Though LMWH was useless in HSOS following HSCT, few clinical trials showed its effectiveness in PA-induced HSOS [7, 8] LMWH was suggested as the only first-line drug to treat PA-induced HSOS by the Chinese Society of Gastroenterology Committee of Hepatobiliary Disease [6]. Therefore LMWH served as a positive control of defibrotide in our study.

Survival was ‘hard’ end-point when evaluating the effect of a drug. So we needed a rat HSOS model whose survival time could be measured. There had been several HSOS models before [18–20], but none of them met our requirements. DeLeve developed the first model in 1999, in which 160 mg/kg MCT garage was used to induce HSOS in rats. In that model, all rats survived longer than 10 days after garage and the survival time was not reported [20]. In the following studies, MCT garage at a dose of 90 mg/kg was adopted and the rats were sacrificed within 48 h to evaluate the effectiveness of certain drug by pathology assessment [18, 19, 21]. In this study, we explored a high dose of MCT (230 mg/kg) as LD50 with a 7-day survival rate of  43.7% (7/16). The manifestation and pathology features proved that the model was successfully built: increased liver function and WBC count, liver endothelial injury, necrosis, bleeding, fibrosis, etc. We believed the model was more suitable for drug effect evaluation than tradition models.

Defibrotide has been studied as an anti-coagulation and endothelial protection agent in different rat models. In those studies, defibrotide was given by different routes of administration and at different dosages in different animals [22–24]. For example, Kim et al. [25] examined the effect of defibrotide on liver ischemia-reperfusion injury. In the experiment, an intravenous bolus of defibrotide was given at the dose of 10 mg/kg. In our research, the dose of defibrotide was calculated according to the drug instruction. Defibrotide was given at a dose of 50 mg/kg/d intravenously every day, which was significantly higher than previous studies. We found that defibrotide was more effective than LMWH almost in all aspect.

SESs injury induced release of TNF-α and IL-1β followed by PAI-1 expression elevation. PAI-1 was a biomarker of endothelial injury, which increased significantly in HSOS [16, 26]. Our research found that plasma level of TNF-α and PAI-1 were significantly higher in the model group than the control group and decreased in group E, which indicated protection of SEC by defibrotide.

## Conclusion

Our research first demonstrated the effectiveness of defibrotide on MCT-induced HSOS which indicating it’s candidate role for clinical use in the future.

There were also some limitations of the study. First, the results should be further confirmed in different animal models, such as seneciphylline, another kind of PA. Second, we didn’t study the mechanism thoroughly of defibrotide on the model. Third, defibrotide should be administered intravenously every 6 h according to the introduction. But we gave defibrotide once daily in the research. The administration method should be adjusted to find the best method in future studies. Nevertheless, our research provided a new choice for HSOS treatment after poisoning by PA.

## Data Availability

The datasets used and/or analyzed during the current study are available from the corresponding author on reasonable request.

## Declarations

### Author information

Zhenli Liu, lzl910624@126.com. Shan Liang, cat216@163.com. Xinhuan Wei, syweixinhuan@163.com. Xiaofei Du, duxiaofei@ccmu.edu.cn. Jing Zhang, zjyouan@ccmu.edu.cn.

## Abbreviations

CV: Central Venule
CR: Complete Response
H&E: Hematoxylin and Eosin
HSCT: Hematopoietic Stem Cell Transplantation
HSOS: Hepatic Sinusoidal Obstruction Syndrome
HVOD: Hepatic Veno-Occlusive Disease
LMWH: Low Molecular Weight Heparin
MCT: Monocrotaline
PA: Pyrrolizidine Alkaloids
TNF-α: Tumor Necrosis Factor-Α
PAI-1: Plasminogen Activator Inhibitor-1
